# Phytosynthesis of Ag@AgCl nanoparticles using two types of bionanotechnological protocols exhibiting unique antimicrobial, antioxidant, and anti-inflammatory properties

**DOI:** 10.3389/fbioe.2026.1743887

**Published:** 2026-04-15

**Authors:** Yoshinee Doongoor, Joyce G. Soulange, Marek Kolenčík

**Affiliations:** 1 Faculty of Agriculture, University of Mauritius, Reduit, Mauritius; 2 Institute of Agrochemistry and Soil Science, Faculty of Agrobiology and Food Resources, Slovak University of Agriculture in Nitra, Nitra, Slovakia

**Keywords:** Ag@AgCl nanoparticles, anti-inflammatory, antimicrobial activity, antioxidant, bionanotechnology, *Psiadia terebinthina*

## Abstract

**Introduction:**

The synthesis of nanomaterials aims to integrate scientific innovation with a commitment to address the UN’s Sustainable Development Goals (SDGs). Our study reports two biotechnological protocols for the synthesis of colloidal Ag@ AgCl nanoparticles (Ag@AgCl-NPs) using the endemic Mauritian herb *Psiadia terebinthina* via microwave extraction (ME) and heat extraction (HE) methods.

**Methods:**

The Ag-based nanoparticles (NPs) containing colloids and extracts were tested for antimicrobial, antioxidant, and anti-inflammatory activities. The successful bottom-up phytosynthesis of Ag@AgCl-NPs was characterized by transmission electron microscopy (TEM) with chemical verification using energy-dispersive X-ray spectroscopy (EDS), crystallinity assessment using X-ray diffraction (XRD), UV–VIS and Fourier transform-infrared (FT-IR) spectroscopy, and investigation of colloidal properties. Green synthesis approaches favored NP formation and colloidal stability. Antimicrobial activity was tested against Gram-positive (G+) and Gram-negative (G−) bacteria and fungi using the broth microdilution assay. Antioxidant activity was determined by the 2,2-diphenyl-1-picrylhydrazyl (DPPH) method, while anti-inflammatory activity was examined using the bovine serum albumin (BSA) test.

**Results:**

Our results confirmed the successful green bionanotechnological synthesis of Ag@AgCl-NPs with varying relative mass ratios, exhibiting spherical morphology and good crystallinity. Additionally, both colloids with Ag@AgCl-NPs exhibited exceptional synergistic antimicrobial effects against highly resistant G+ and G− bacterial pathogens and the yeast *Candida albicans*. They also demonstrated significant anti-inflammatory activity compared to ME or HE extracts, which, surprisingly, showed greater antioxidant activity.

**Discussion:**

Thus, eco-friendly phytosynthesis from *Psiadia terebinthina* extracts yielded multifunctional hybrid products with potential utility in pharmacology, medicine, and toxicology.

## 1 Introduction

Biotechnological and bioengineering research spans a wide range of academic fields, with functional biomimicry and bionics providing innovative solutions through various types of biomaterials (Hwang et al., 2015). Bionanotechnology focuses on producing stable metal-containing nanoparticles (M-NPs) using accessible biomass or metabolites from microorganisms, such as bacteria (Singh et al., 2015) and fungi (Rahimi et al., 2016; Šebesta et al., 2022), and through plant-based synthesis known as phytosynthesis (Husen and Siddiqi, 2014; Konvičková et al., 2018).

Generally, NPs are materials that must be less than 100 nm in all three dimensions (Grzelczak et al., 2010), characterized by a high surface-to-volume ratio that confers distinct physical, chemical, and biological properties in various environments (Saleh, 2020; Šebesta et al., 2017).

Bottom–up M-NP bio-formation, unlike top–down modification (Tripathy et al., 2023), relies on an atom-by-atom “self-assembly” with the functional participation of biomolecules, secondary metabolites, and plant phytochemicals (Grzelczak et al., 2010; Velgosova et al., 2024). However, key questions remain regarding how to obtain functional biomolecules and the stability of such prepared M-NP colloids because different extraction methods can reduce or modify their yield, activity, and overall effectiveness. Many chemical methods exist, but the literature lacks comparisons of biological *versus* physical extraction for M-NP formation, function, and stability.

One source of biomass is unique plants, which, through synergistic M-NP activity, could enhance their medical and pharmaceutical effects. We have selected *Psiadia terebinthina* A.J Scott, an endemic Asteraceae from Mauritius (Marie et al., 2006), which has been used in the form of “pure” extracts and essential oils for traditional medicinal purposes, including treating pulmonary infections, antimicrobial resistance, and oxidative stress (Gurib-Fakim et al., 1997; Masoumian and Zandi, 2017). A promising approach involves incorporating antiseptic Ag-NPs or AgCl-NPs, which are widely used in medicine, pharmaceuticals, cosmetics, textiles, food packaging, electronics, energy, and construction for their antiviral and antimicrobial properties (Beyene et al., 2017; Eltayeb et al., 2025). For broader applications in bioengineering, the potential anti-inflammatory and antioxidant effects of phytochemicals acting synergistically along with Ag-based NPs, rather than “pure” extracts, which mitigate harmful free radicals, remain largely unexplored and unreported (Shin et al., 2020; Wong et al., 2009).

Therefore, our study aimed to compare microwave (ME) and heat water extraction (HE) of *Psiadia terebinthina* on the nucleation, formation, and colloidal stabilization of Ag@AgCl-NPs. The resulting extracts and both Ag@AgCl-NP colloids were evaluated for antibacterial, antioxidant, and anti-inflammatory activities, highlighting their biotechnological potential.

## 2 Materials and methods

### 2.1 Phytochemical extraction of functional metabolites using physical and heat methods

The first step of the biotechnological protocol involved the collection, identification, and authentication of mature *Psiadia terebinthina* leaves from the University of Mauritius herbarium at Réduit with the accession number MAU 0016420.

The leaves were then rinsed with distilled water to remove impurities, shade-dried until a constant weight was achieved, and ground into a fine powder. This powder was mixed with distilled water at a ratio of 1:20 (g/v) and heated for 15 min at 60 °C–70 °C (Jalab et al., 2021). This variant was designated as heat extraction (HE). For the microwave-treatment procedure (known as microwave extraction, ME), the powdered leaves were mixed with distilled water at a ratio of 1:30 (g/v) and subjected to microwave heating for 2 min (8 s on and 15 s off to prevent boiling) using a 1400 W household microwave oven (Mohanta et al., 2017). The filtered extracts were stored at 4 °C for future use.

Finally, the HE extract was mixed with 2 mM AgNO_3_ in a 1:9 (v/v) ratio (designated as HE Ag@AgCl-NPs), and a similar procedure was applied to the microwaved extract (ME Ag@AgCl-NPs). Changes in the color of the colloids indicated the reduction and the production of phytosynthesized Ag-containing NPs (Jalab et al., 2021), which were then centrifuged at 10,000 rpm for 22 min at 4 °C (Mohanta et al., 2017).

### 2.2 Identification and characterization of phytosynthesized silver-containing nanoparticles

#### 2.2.1 Scanning transmission electron microscopy with energy-dispersive X-ray spectroscopy

The morphology and particle size distribution of both Ag@AgCl-NPs were investigated using the scanning transmission electron microscope (STEM) JEOL JSM-7610F Plus (Tokyo, Japan) with a Schottky cathode at 30 keV in a high-vacuum chamber. Samples were placed on Formvar-coated copper grids. Elemental composition analysis and mapping were performed using the energy-dispersive X-ray spectroscope (EDS) AZTEC Ultima Max 65 (Oxford Instruments, Abingdon, United Kingdom).

#### 2.2.2 X-ray diffraction analysis

X-ray diffraction (XRD) analysis was employed to evaluate the crystallinity and structural parameters of both phytosynthesized Ag@AgCl-NPs. XRD measurement was performed using a Bruker X8 DISCOVER diffractometer (Bruker, MA, United States) under the following conditions: 12 kW (40 kV, 300 mA) with a Cu anode. The data were used to calculate unit cell parameters with TOPAS 3.0 software (Bruker, MA, United States).

#### 2.2.3 UV–VIS spectroscopy

Both colloids containing Ag@AgCl-NPs were subjected to spectrophotometric analysis in the range of 200–700 nm at intervals of 60 min, 24 h, 48 h, and 1 week, following the method described by Balashanmugam and Kalaichelvan (2015).

#### 2.2.4 Fourier-transform infrared spectroscopy

The analysis of the dried sample from the Ag@AgCl-NP colloids was conducted using a highly compact Bruker ALPHA FT-IR spectrometer. The measurement conditions and sample transmittance were processed using OPUS software (Balashanmugam and Kalaichelvan, 2015).

#### 2.2.5 Assessment of selected colloidal properties of Ag@AgCl nanoparticles

The examined colloidal properties, that is, the zeta potential (*ζ*-potential) and conductivity of nanoparticle-containing colloids, were determined using a nanoPartica SZ-100 analyzer (Horiba, Kyoto, Japan). Measurements were performed using a microprocessor-controlled cell, and the zeta potential was calculated directly under laboratory conditions employing the generalized Smoluchowski equation.

### 2.3 Analysis of major phytochemicals obtained from *Psiadia terebinthina* leaves using heat- and microwave-treated methods

The HE and ME extracts of *P. terebinthina* were first screened for phytochemicals using standard methods (Harborne, 1998). To determine the qualitative content of functional groups involved in the Ag@AgCl-NP formation, the presence of phytochemicals such as coumarins, flavonoids, carbohydrates, diterpenes (Ashvin Godghate et al., 2012), alkaloids (Hultin and Torssell, 1965), terpenoids (Misbah Manzoor et al., 2013), tannins and steroids (Bhajan et al., 2022), and phenols (Batool et al., 2019) was assessed.

The phenolic contents were determined using the Folin–Ciocalteu assay. Gallic acid (0.1–0.6 mg/L) served as the standard, and distilled water served as the blank. For phenolic estimation, 100 µL of sample or standard was mixed with 2.9 mL of distilled water and 5 mL of the Folin–Ciocalteu reagent. After 5 min, 2 mL of 20% sodium carbonate was added, and the mixture was incubated at room temperature for 1 h before measuring absorbance at 650 nm (Sivakumar, 2021).

For tannin estimation, 100 µL of sample or standard was combined with 7.5 mL of distilled water, 0.5 mL of Folin–Ciocalteu reagent, and 1 mL of 35% sodium carbonate and then diluted to 10 mL. Absorbance was measured at 725 nm (Roy et al., 2018). All measurements were performed in triplicate.

### 2.4 Comparison of antioxidant activity

The antioxidant activity of HE-Ag@AgCl-NPs, ME-Ag@AgCl-NPs, and their respective HE and ME extracts was evaluated using the DPPH radical scavenging assay (Keshari et al., 2020). Vitamin C served as the standard for constructing the calibration curve. Test samples (200–1,000 μg/mL) of all NP colloids and extracts were prepared. A 0.3 mM DPPH solution in methanol was freshly prepared, and 2 mL of this solution was mixed with 2 mL of each test sample and vortexed. The control contained only DPPH solution, with methanol as the blank. Mixtures were incubated in the dark for 30 min, and absorbance was recorded at 517 nm. The percentage inhibition was calculated using the following equation:

$$\text{DPPH radical scavenging activity }\left(\%\right)=\left(\frac{\left[\text{Abs}\;\text{control}-\text{Abs}\;\text{sample}\right]}{\text{Abs}}\text{control}\right)*\;100.$$

### 2.5 Comparison of anti-inflammatory activity

The anti-inflammatory activity of HE Ag@AgCl-NPs, ME Ag@AgCl-NPs, and their equivalent pure extracts was analyzed using the bovine serum albumin (BSA) denaturation inhibition assay (Kaliammal et al., 2021; Vernekar and Taranath, 2022). Diclofenac sodium was used as the standard for preparing the calibration curve. Test samples of both Ag@AgCl-NP colloids and the extracts (200–1,000 μg/mL) were prepared. A 1% BSA solution was made by dissolving BSA in distilled water (1:100, w/v). Phosphate-buffered saline (PBS) was prepared and adjusted to pH = 6.4 using 1 M HCl. The reaction mixture consisted of 0.2 mL BSA, 2.8 mL PBS, and 2 mL of the test sample. All measurements were carried out in triplicate. The mixtures were incubated at 37 °C and then heated in a water bath at 70 °C for 10–15 min. After cooling, absorbance was measured at 660 nm. The percentage inhibition of protein denaturation was calculated using the following formula:

$$\text{Percentage inhibition }\left(\%\right)=\left(\frac{\left[\text{Abs}\;\text{control}-\text{Abs}\;\text{sample}\right]}{\text{Abs}}\text{control}\right)*\;100.$$

### 2.6 Comparison of antimicrobial activity

Antibacterial activity was tested against Gram-positive bacteria, *Staphylococcus aureus* (ATCC 25923), and Gram-negative bacteria such as *Escherichia coli* (ATCC 25922) and *Pseudomonas aeruginosa* (ATCC 27853), while antifungal activity was assessed against *Candida albicans* (ATCC 10231).

The well micro-dilution assay (Eloff, 1998) was used to determine the minimum inhibitory concentration (MIC) of the extracts and the Ag@AgCl-NPs. The positive control was 100 μl of sterile distilled water and 100 µl of nutrient/Mueller Hinton broth, and the negative control was 100 μl of sterile distilled water and 100 µl of chloramphenicol/nystatin. A 100 μl aliquot of microorganism (bacterial/fungal culture) with absorbance 0.400–0.600 nm at a wavelength of 600 nm was dispensed in all the wells containing the solution. The microplates were incubated overnight at 37 °C. The 0.2 mg/ml INT (p-iodonitrotetrazolium violet) solution was freshly prepared, and 40 µl of INT solution was added to every well, after which the microplates were incubated for approximately 30 min at 37 °C. After 30 min, the development of a pink color in any well indicated microbial growth.

### 2.7 Statistical analysis

Microsoft Excel 2016 was used to process all experimental data to obtain the mean and standard deviation. Standard curves were plotted using linear regression to obtain the best-fit line. Jamovi software was used to perform Tukey’s pairwise comparisons (ANOVA). A *p*-value of less than 0.05 obtained for the ANOVA was considered statistically significant. Pearson’s correlation coefficient was processed in the Minitab software to determine the correlation between quantitative phytochemical assays and the bioassays.

## 3 Results and discussion

### 3.1 Formation and colloidal stabilization of Ag@AgCl-NPs employing two bionanotechnological routes

Modern bionanotechnological approaches for M-NP synthesis exploit diverse biomass sources. Plant-mediated phytosynthesis offers sustainable species diversity, simple preparation, biocompatibility, and the simultaneous reduction of metal ions to NPs with their *in situ* stabilization (Abdel-Hafeez and Abdel-Goad, 2025; Alharbi et al., 2022).

An open academic question remains as to which extraction technique is more effective in preserving the quality and quantity of phytochemicals obtained through extraction procedures (Shen et al., 2023; Zhang et al., 2018). In this context, each extraction method provides distinct technological, economic, and environmental advantages (Alharbi et al., 2022). In our case, microwaved-derived and heat-derived extracts of *Psiadia terebinthina* (Table 1) were successfully equipped for the Ag@AgCl-NP phytosynthesis, yielding varying amounts of phytochemicals.

**TABLE 1:** Comparative phytochemical yields of *Psiadia terebinthina* extracts prepared using HE and ME extraction methods.

Sample of *Psiadia terebinthina*	Heat extract (HE)	Microwaved extract (ME)
Average percentage phytochemicals (%)	7.04 ± 1.41	10.32 ± 2.94

A difference was observed, with the ME method yielding a statistically significant (*p* < 0.05) higher amount of extracted phytochemicals than the HE method, likely due to the kinetically accelerated disruption of plant cell walls and tissues (Kishimoto, 2022). However, no study has reported whether phytochemicals obtained through ME exhibit similar or different bioactive mode-of-action as those obtained by low temperature HE (Routray and Orsat, 2012).

There is some evidence that the relative proportions of phytochemicals remained consistent across both extraction procedures (Table 2), confirming the presence of phenols, coumarins, flavonoids, and terpenes in the leaves of *Psiadia terebinthina*, as also settled by Marie et al. (2006).

**TABLE 2:** Qualitative phytochemical profiles of *Psiadia terebinthina* extracts obtained by heating and microwave-assisted treatment.

Phytochemical test	Heat extract (HE)	Microwave extract (ME)
Coumarins	++	++
Flavonoids	++	++
Alkaloids	+	+
Terpenoids	++	++
Tannins (hydrolyzable)	++	++
Steroids	−	−
Carbohydrates	+	+
Phenols	++	++
Diterpenes	++	++

++, abundantly present; +, traces present; −, absent.

The most statistically significant differences between the extracts were observed in the total phenolic content (TPC) and total tannin content (TTC) (Table 3), preferring microwave extraction. Comparison of TPC and TTC showed a great decrease after the phytosynthesis of Ag@AgCl-NPs in both extracts, indicating the active role of these phytochemicals in NP production.

**TABLE 3:** Comparative analysis of total phenolic and tannin contents in *Psiadia terebinthina* extracts and their corresponding phytosynthesized Ag@AgCl nanoparticles after phytosynthesis.

Samples	Total phenolic content (TPC) value (mg GAE/g extract)	Total tannin content (TTC) value (mg GAE/g extract)
Heat extract (HE)	284.03 ± 22.8^b^	219.37 ± 23.13^b^
HE Ag@AgCl-NPs	51.02 ± 14.11^a^	41.96 ± 7.91^a^
Microwave extract (ME)	378.67 ± 32.7^c^	336.10 ± 5.31^c^
ME Ag@AgCl-NPs	80.53 ± 54.85^a^	76.26 ± 5.51^a^

Different lowercase letter superscripts represent a significant difference between samples (*p* < 0.05).

In general, the formation of M-NPs begins with nucleation processes, followed by the growth and development of dominant shapes, during which NP stabilization is taking place by reaching a current equilibrium of all relevant components in the colloids (Konvičková et al., 2018). In our case, Ag^+^ ions were reduced to Ag^0^ NPs, while Ag^+^ was transformed into the thermodynamically stable AgCl-NP phase in both phytosynthesis routes. After the initial formation of Ag@AgCl-NPs, a diffuse double-layer coating develops, whose equilibrium state and functional properties are governed by the ionic strength of the surrounding bulk phase.

However, slight deviations in the functional groups of organic molecules and phytochemicals within the colloids involved in phytosynthesis led to several minor differences in the physicochemical properties of the NPs.

**FIGURE 1:**
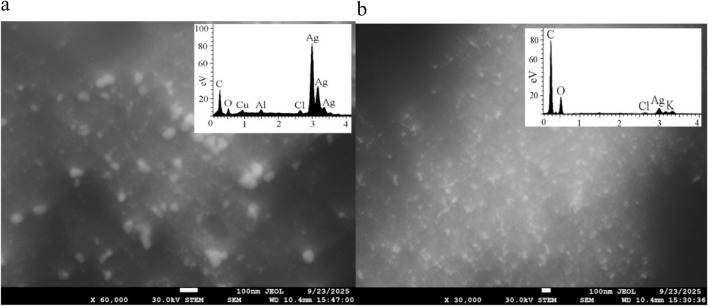
Morphology of Ag@AgCl-NPs after phytosynthesis using the *Psiadia terebinthina* microwaved extract **(a)** and heated extract **(b)** as observed in scanning transmission electron microscopy (STEM) mode. The figure includes a chemical verification performed by EDS.

Based on FT-IR analysis, minimal differences between the ME and HE extracts were observed (Figure 1). In both colloids, the presence of hydroxyl groups was identified (Figure 1), commonly found in phenols, which adsorb on the surface of Ag-NPs and stabilize the flower-extracted solution (Jini et al., 2022; Johnson et al., 2018) or could induce the formation of Ag@AgCl-NPs (Khatun et al., 2024). As reported by Wan Mat Khalir et al. (2020), terpenoids bearing aromatic functional groups that facilitate Ag^0^ NP formation were likewise present in the ME and HE extracts. The presence of secondary and tertiary alcohols, typically found in carbohydrates (Ebrahiminezhad et al., 2016), as in our results, could play a significant role as capping agents for stabilizing Ag-NPs in the colloids synchronically with the formation of Ag@AgCl-NPs (Okaiyeto et al., 2019). Various functional groups and their exposed active bonds observed in both extracts are the mechanisms primarily responsible for the production of Ag-related crystalline phases, acting as electron donors for Ag-NP or AgCl-NP phytosynthesis (Ebrahiminezhad et al., 2016; Okaiyeto et al., 2019). Additionally, these groups improve colloidal stability through the formation of a diffuse double layer serving as a functional coating (Konvičková et al., 2018). These groups may exert an influence as a non-colloidal aggregation agent or support electro-repulsive forces. In our study, FT-IR analysis also confirmed the insignificant presence of bonds corresponding to C-Cl and C-Br, identified for the first time in the *Psiadia terebinthina* extract.

**FIGURE 2:**
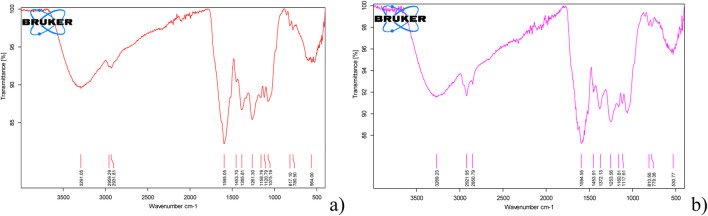
FT-IR spectra of different types of functional phytochemicals from **(a)** heat extraction and **(b)** microwave extraction of *Psiadia terebinthina* and phytosynthesized Ag@AgCl-NP colloids.

In the case of the ME extract, STEM analysis revealed predominantly spherical and occasionally spheroidal morphology of Ag@AgCl-NPs ranging from 30 nm to 100 nm in size, with occasional aggregation (Figure 2a). EDS analysis confirmed that the particles consisted mainly of Ag, with minor amounts of Cl, C, and O. The presence of Cu and Al was likely due to signals from the sample holder and copper grid. The extract obtained by heat treatment, such as the ME extract, also exhibited predominantly spherical morphology of NPs within the size range of 50–100 nm (Figure 2b). Chemically, the NPs contained elements such as Ag, Cl, C, K, and O, corresponding both to Ag and AgCl-NPs and to the functional groups responsible for their phytosynthesis.

Somewhat unexpected results were observed during phytosynthesis. The ME extract produced a lower Ag:AgCl mass ratio (29:71) than the heated extract (45:55). Slight differences were observed in the particle size dimensions of the internal crystallites of Ag and AgCl-NPs, that is, L_vol-IB_, in both phytosynthesis routes (Table 4). These variations likely arise from differences in phytochemical composition (Tables 1–3), as also reported by Khatun et al. (2024), whereas structural parameters such as symmetry, space group, and *a*-axis remained essentially identical.

**TABLE 4:** Comparison of the Ag@AgCl-NP colloids prepared from HE and ME extracts, their symmetry, and other crystallographic parameters.

​	ME Ag@AgCl-NPs		HE Ag@AgCl-NPs	
Obtained nanoparticles	Ag	AgCl (chloratgyrite)	Ag	AgCl (chloratgyrite)
Mass ratio of the obtained minerals	29%	71%	45%	55%
Symmetry	Cubic	Cubic	Cubic	Cubic
*a*-axes	4.087 ± 0.001 Å	5.5548 ± 0.0006 Å	4.085 ± 0.002 Å	5.551 ± 0.0006 Å
Space group	Fm-3m	Fm-3m	Fm-3m	Fm-3m
L_vol_-IB (nanometers)*	5.9 ± 0.3	11.2 ± 0.3	3.9 ± 0.2	12.0 ± 0.3

* Calculated from the obtained X-ray diffraction data using TOPAS software.

**FIGURE 3:**
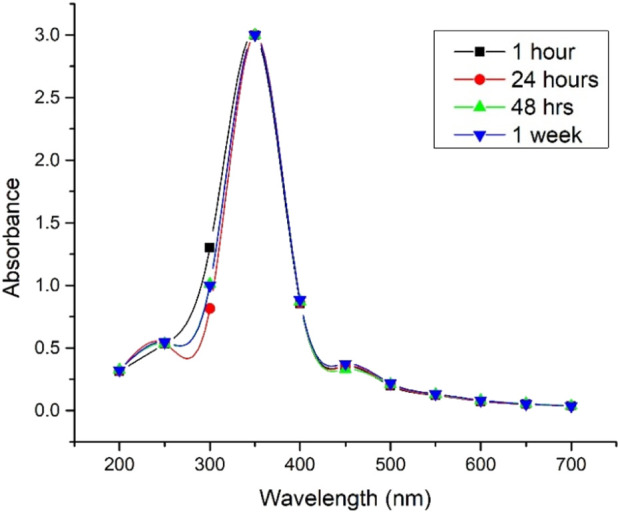
UV–VIS spectra of Ag@AgCl nanoparticle colloids phytosynthesized from heated *Psiadia terebinthina* extracts over time under natural daylight exposure.

**FIGURE 4:**
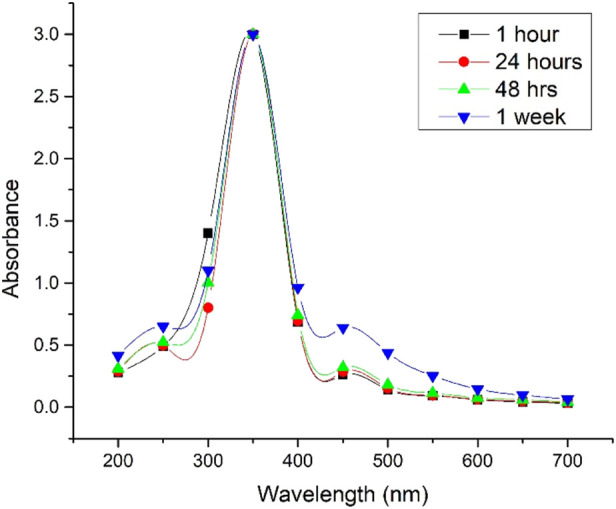
UV–Vis spectra of Ag@AgCl nanoparticle colloids phytosynthesized from microwaved *Psiadia terebinthina* extracts over time under natural daylight exposure.

In both cases, the synthesized Ag@AgCl-NPs exhibited UV–VIS absorption peaks at approximately 350 nm (Figures 3, 4). Kelly et al. (2003) reported this peak for spherical Ag-NPs with a size of approximately 60 nm, which has relatively good agreement with our findings. Other studies have also confirmed that AgCl shows absorption at this wavelength, depending on its size, distribution, and morphology (Cheon et al., 2018).

In the context of key colloidal properties, including *ζ*-potential and electrical conductivity, only a slight difference was observed between HE Ag@AgCl-NPs and ME Ag@AgCl-NPs. ME Ag@AgCl-NPs exhibited a higher *ζ*-potential (24.1 ± 1.2 mV), indicating relatively more stable colloidal properties with moderate electrostatic repulsion than HE Ag@AgCl-NPs, which showed a lower electrostatic value (16.2 ± 1.9 mV). However, this colloid displayed higher electrical conductivity (Table 5), which could theoretically correspond to a greater number of functionally ionic-active groups of phytochemicals (Tables 1–3). Colloidal stability is generally considered sufficient when the *ζ*-potential exceeds +20 mV or more than −20 mV for similarly charged nanoparticles (Mostafa et al., 2024).

**TABLE 5:** Evaluation of zeta potential (*ζ*-potential) and electrical conductivity of both Ag@AgCl-NP colloids phytosynthesized using two different extraction methods.

Samples	Zeta potential (mV)	Conductivity (mS/cm)
HE Ag@AgCl-NPs	−24.1 ± 1.2	0.45 ± 0.01
ME Ag@AgCl-NPs	−16.2 ± 1.9	0.80 ± 0.80

In general, one of the main risks associated with biologically and phytochemically prepared extracts forming colloids, particularly those containing metallic NPs, including Ag-NP, is their colloidal stability. This stability can be affected by changes in thermodynamic factors, including temperature, light exposure, or other externally activated radiation or electromagnetic energy (Kolenčík et al., 2014; Poudel et al., 2022). In our case, UV–VIS spectra after 1 week of exposure to natural daylight showed no significant shift at 350 nm, indicating relatively good stability and low reactivity of the *Psiadia terebinthina* colloids (Figures 3, 4).

### 3.2 Comparison of antimicrobial activity of phytosynthetized Ag-containing NPs and extracts against selected bacterial and fungal species

The results of the antimicrobial activity based on the minimum inhibitory concentration assay (Table 6) indicate that both phytosynthesized Ag@AgCl-NP colloids exhibited greater efficacy than the corresponding pure extracts. The high antimicrobial effectiveness of both Ag@AgCl-NP colloids was nearly comparable to the tested antibacterial pharmacological standards, particularly chloramphenicol for both G+ and G− bacteria and nystatin for the fungus *Candida albicans*.

**TABLE 6:** Comparison of antimicrobial activity based on MIC of two colloidal Ag@AgCl-NPs and the HE and ME extracts of *Psiadia terebinthina* against Gram-positive and Gram-negative bacteria and fungi.

Minimum inhibitory concentration (mg/ml)					
Name of microorganism	Heat extract (HE)	HE Ag@AgCl-NPs	Microwave extract (ME)	ME Ag@AgCl-NPs	Control
*Escherichia coli*	2.06	0.02	1.10	0.078	0.0038*
*Pseudomonas aeruginosa*	1.05	0.02	1.78	0.16	0.0038*
*Staphylococcus aureus*	1.05	0.16	1.10	0.078	0.0019*
*Candida albicans*	8.43	0.02	3.53	0.04	0.0016**

*Chloramphenicol as the standardized control chemical agent for G+ and G− bacteria in the concentration range of 0.0019 ≤ MIC ≤0.0038. **Nystatin as the standardized control chemical agent for fungi with a concentration of 0.0016 g/mL.

The enhanced antimicrobial activity of both Ag@AgCl-NP colloids can be attributed to the physicochemical properties of the NPs and the functional groups from the original extract forming the colloid (Tables 1–3). Initial interactions involve the attachment, adsorption, and uptake of Ag@AgCl-NPs at the cell walls of the tested pathogens. Subsequently, their nano-scale action facilitates penetration, influenced by charged ions on the pathogen surfaces (Peiris et al., 2017; Schreurs and Rosenberg, 1982).

The kinetically driven toxic activity of Ag@AgCl-NP colloids is mediated by the generation of ROS species (Rahimi et al., 2016). This silver species is known for its toxic effects on a wide range of microorganisms (Beer et al., 2012), including Gram-positive bacteria *Staphylococcus aureus* or Gram-negative bacteria *Escherichia coli* (Konvičková et al., 2018). Oxidative damage to bacterial membranes, including protein and DNA disruption, was also observed for Ag@AgCl NPs synthesized from *Jatropha* seed extract in real petrochemical wastewater, resulting in a 92% reduction in bacterial assemblages (Abdel-Hafeez and Abdel-Goad, 2025).

Although the effect of Ag^+^ ions on microorganisms was not directly assessed in our study, a certain degree of thermodynamic metastability of the Ag^0^-NPs *versus* AgCl nanophase is assumed, where released Ag^+^ ions could directly integrate into degradation mechanisms of the extracellular polymeric matrix in biofilms, inhibition of protein synthesis, or other potential toxic modes of action (Rodrigues et al., 2024). The mass ratio between Ag and AgCl-NPs (Table 4) should be taken into consideration as it may partially explain the observed deviations in antibacterial and antifungal effectiveness (Table 6).

For fungal targets (Table 6), Atef et al. (2013) reported that Ag-NPs disrupt fungal membranes by forming pits in the cell walls, leading to leakage of cellular components and ultimately cell death. Antifungal activity against *Candida tropicalis*, along with antibiofilm effects, was also observed for Ag@AgCl-NPs synthesized from the tropical tree *Azadirachta indica* (Al Aboody, 2019). Our results are consistent with a significant level of antifungal activity observed for phytosynthesized Ag@AgCl-NPs; however, this effect was achieved at a higher applied dose than that reported by Sharifi-Rad and Pohl (2020). In their study, the MIC of biosynthesized AgCl-NPs prepared using *Pulicaria vulgaris* Gaertn. was evaluated against *Candida glabrata* and *Candida albicans* at lower concentrations ranging from 40 µg/mL to 60 μg/mL.

Similarly to our particle size distribution, Abed-Alwahed and Al-Baqi (2020) reported biosynthesized Ag-NPs with sizes ranging from 30 nm to 60 nm, which reduced the resistance of *Candida albicans* isolates by decreasing the expression of resistance-related genes, such as *ERG11*, through various mechanisms when compared with commercial antifungal agents.

The synergistic antimicrobial effect may also be associated with extracts from *Psiadia terebinthina*, primarily due to the presence of essential oils such as pinene, β-myrcene, and eugenol, which are active against Gram-negative cosmopolitan bacteria, including *Pseudomonas* species (Govinden-Soulange et al., 2004). Additionally, FT-IR analysis indicated the presence of reactive intermediates in the solution, with peaks at 790.5–779.3 cm^−1^ (Figure 1), corresponding to alkene groups of diterpenes containing isoprene units (Flamini, 2012; van Hylckama Vlieg et al., 2000). These compounds facilitate enzymatic modifications, underpinning the strong biocidal and fungicidal activity of the extracts.

### 3.3 Comparison of the anti-inflammatory activity of extracts and Ag@AgCl-NPs

**FIGURE 5:**
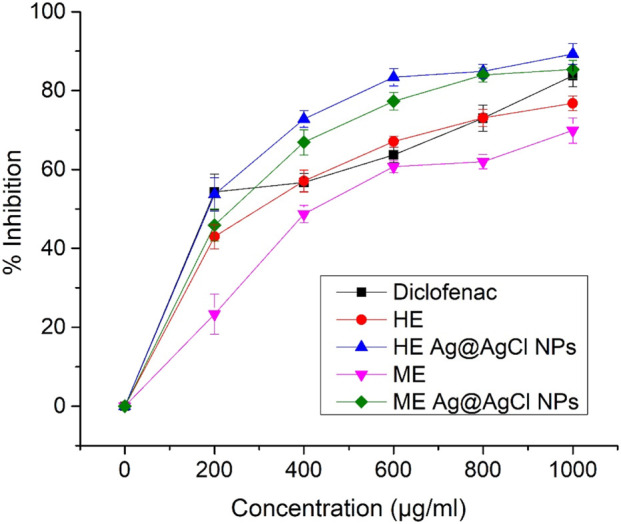
Anti-inflammatory activity measured by the BSA assay, expressed as % inhibition at increasing concentrations of diclofenac (control), HE and ME *Psiadia terebinthina* extracts, and both Ag@AgCl-NP colloids.

The results of the anti-inflammatory activity assay (AIA) indicate that all tested concentrations of Ag@AgCl-NP colloids and HE or ME extracts (0, 200 µg/ml, 400 µg/ml, 600 µg/ml, 800 µg/ml, and 1,000 μg/ml) exhibited a similar time-related statistical trend. However, the most effective AIA was observed at the final concentration of 1,000 μg/ml, with HE Ag@AgCl-NP colloids showing greater efficacy than ME Ag@AgCl-NPs, both achieving 85% activity compared to the tested standard diclofenac sodium (83%). Significantly lower efficacy (p < 0.05) was observed for the heat-derived extract (76.8%) and the microwaved extract (69.9%) (Figure 5).

The *in vitro* anti-inflammatory preliminary assays reveal the potent effect based on the % of BSA inhibition evaluated with IC_50_ values: HE Ag@AgCl NPs > ME Ag@AgCl NPs > control (diclofenac sodium) > HE extract > ME extract (Supplementary Table 7).

In our study, phytosynthesized Ag@AgCl-NPs produced from ME and HE extracts exhibited potent anti-inflammatory efficacy compared to other treatments. This effect was observed at higher concentration levels (>320 μg/mL) than those used by Berihu et al. (2021). The anti-inflammatory and cytotoxic activities of Ag-NPs synthesized via a green chemistry route using *Terminalia brownii* demonstrate a dose-dependent *in vitro* inhibition of nitric oxide (NO) production and the inflammatory mediator prostaglandin E_2_ (PGE_2_), which correspond to IC_50_ values ∼32 μg/mL and ∼67 μg/mL, respectively.

An interesting finding in our study is the relatively ineffective anti-inflammatory efficacy of the ME and HE extracts. However, further cell-based assays are required to confirm the superior anti-inflammatory activity observed in phytosynthesized Ag@AgCl-NPs. The most likely explanation is the efficiency of extraction, which releases organic molecules, or differences in the extract pretreatment route, particularly ethanol-based extraction, which may enhance synergistic antiseptic impact (Makchuchit et al., 2010).

Preliminary studies conducted by Wong et al. (2009) attribute the effect of Ag-NPs in promoting fast healing in animal models of thermal injury to their ability to reduce wound inflammation and control the production of fibrogenic cytokines. Phytochemicals, such as phenols and terpenoids, also play a role in preventing inflammatory diseases by regulating various signaling pathways (Shin et al., 2020).

Research by Jabir et al. (2021) reported that Ag-NPs principally could stimulate phagocytosis and neutralize inflammatory microorganisms, while herbal extracts may enhance therapeutic strategies through the body’s immune response, particularly in affected tissues. Ag-NPs combined with medicinal plant extracts can inhibit the activity of cyclooxygenase (COX-2) and lipoxygenase (5-LOX) as potential drug delivery system administrators involved in inflammation (Ilahi et al., 2021). Kang et al. (2018) demonstrated that Au-NPs and AgCl-NPs functionalized with *Crataegus pinnatifida* fruit extract act as novel anti-inflammatory agents, largely due to their high flavonoid content. The study showed that these NPs effectively reduced the production of inflammatory mediators, including nitric oxide and prostaglandin E2, in lipopolysaccharide-stimulated RAW264.7 cells. Nevertheless, comprehensive research combining antiseptic nanometals with medicinal herb extracts possessing strong antioxidant activity remains limited.

### 3.4 Comparison of the antioxidant activity of Ag@AgCl-NP colloids with that of the extracts

**FIGURE 6:**
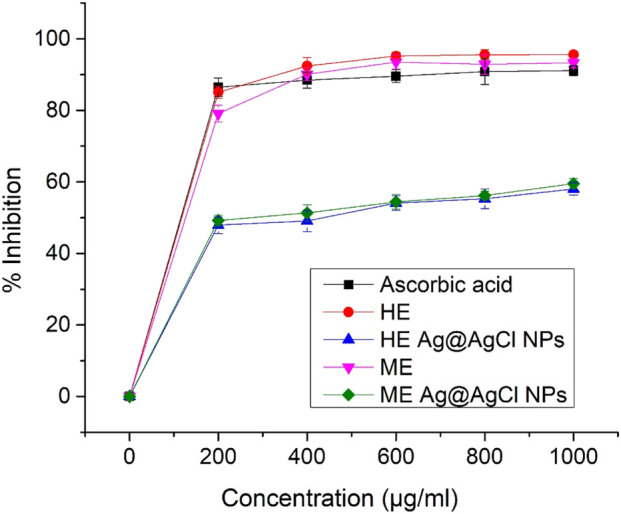
DPPH assay-based antioxidant activity (% inhibition) of ascorbic acid, HE and ME extracts, and both Ag@AgCl-NPs across increasing concentrations.

The results of the DPPH assay (Figure 6) indicate a consistent trend in antioxidant activity across all observed concentrations (0, 200–1,000 μg/ml), with both the extracts and the standard-ascorbic acid demonstrating statistically significant levels. However, these findings do not align with the antimicrobial and anti-inflammatory activities of Ag@AgCl-NP colloids as the HE and ME extracts were more effective, exceeding 93%, than the corresponding ME Ag@AgCl-NP (59.64%) and HE Ag@AgCl-NP (57.5%) colloids. Although Ag-NPs can aid radical scavenging (Bedlovičová et al., 2020), the observed antioxidant activity is mainly due to the phytochemicals in the plant extract, not the NPs themselves (Figure 6).

The antioxidant activity, expressed as EC_50_ values based on the 2,2-diphenyl-1-picrylhydrazyl (DPPH) assay, was observed in the following order: HE extract > ascorbic acid > ME extract > ME Ag@AgCl-NPs > HE Ag@AgCl-NPs (Supplementary Table 8).

In our study, HE and ME extracts exhibited the strongest antioxidant activity, with EC_50_ values of ∼130 μg/mL and ∼160 μg/mL, respectively. In comparison with our results, Makchuchit et al. (2010) reported slight discrepancies in DPPH-based antioxidant activity. This study assessed 18 medicinal plant extracts prepared by maceration, aqueous extraction, and boiling. EC_50_ values generally ranged from ∼7 µg/mL to >100 μg/mL, with *Syzygium aromaticum* exhibiting the strongest activity (∼5 μg/mL) across all extraction procedures. Previous studies using the essential oil of *Psiadia terebinthina* reported good antioxidant activity with an EC_50_ value of 0.931 μg/ml (Mahomoodally et al., 2019). Chemicals such as curcumene, *ß*-pinene, myrcene, isoeugenol, benzyl alcohol, eugenol, methyl eugenol, vanillin, acetovanillone, and limonene were identified in *P. terebinthina* essential oil. They may be involved in its RSA activity (Govinden-Soulange et al., 2004). In the current study, a strong positive correlation was observed between antioxidant activity and total phenolic content (TPC; r = 0.967) and total tannin content (TTC; r = 0.931). This suggests that phenols and tannins are most likely responsible for the antioxidant activity of both extracts and, together with other organic molecules, may contribute to their antioxidant effects (Akintola et al., 2020).

Our results showed promising antioxidant activity, which was also observed in the control standard using ascorbic acid, with an EC_50_ value of ∼137 μg/mL (Supplementary Table 8). In contrast, Aderogba et al. (2007) reported higher antioxidant activity in the DPPH assay for ascorbic acid, with an EC_50_ value of ∼20 μg/mL, indicating stronger radical scavenging capacity.

The results indicate that the lowest antioxidant potential was observed for both extraction procedures, with EC_50_ values exceeding 600 μg/mL. According to DLVO theory, the hypothetically reduced antioxidant activity may have been caused by aggregation of Ag-related NPs (Bedlovičová et al., 2020); however, UV–VIS analysis indicated that our colloids remained stable for 1 week (Figures 3, 4). A similar trend of lower antioxidant activity in biosynthesized Ag-NPs prepared using an aqueous extract of *Agrimonia eupatoria* L. was also reported by Marković et al. (2024). Thus, the evaluation using the DPPH and ABTS assays demonstrated EC_50_ values exceeding 100 μg/mL, also reflecting a comparatively weak antioxidant capacity of the samples.

## 4 Conclusion

Our study reports two efficient, environmentally friendly protocols for phytosynthesizing Ag@AgCl-NPs from the Mauritian endemic *Psiadia terebinthina* using a bottom–up approach.

Microwave-assisted extraction (ME) yielded higher phytochemical levels, particularly phenols and tannins, than heat extraction (HE), influencing functional groups involved in phytosynthesis. These differences in functional groups were slightly reflected in the nucleation, formation, stabilization, and Ag-to-AgCl-NP mass ratio in both colloids. These resulted in the Ag@AgCl-NPs being predominantly spherical (<100 nm), crystalline, and colloidally stable, with moderate *ζ*-potential for HE-formed NPs and lower values for ME-produced NPs.

Both Ag@AgCl-NP colloids showed increased antimicrobial activity against model pathogenic Gram+ and Gram− bacterial species and the fungus *Candida albicans*, compared with both extracts. Moreover, a positive trend of both Ag@AgCl-NP colloids was provided in the BSA assay, indicating anti-inflammatory activity. In contrast, both extracts were more effective in the DPPH assay-based antioxidant activity than the phytosynthesized Ag@AgCl-NP colloids.

Our research confirmed the environmentally friendly metal-nanomaterial phytosynthesis protocols with multispectral biotechnological potential. Further *in vitro* and *in vivo* studies of the biocompatibility profile and cytotoxicity are warranted to investigate safety and potential toxicity for pharmacological exploitation.

## Data Availability

The raw data supporting the conclusions of this article will be made available by the authors, without undue reservation.
