# Supplementary material for: Phytosynthesis of Ag@AgCl nanoparticles using two types of bionanotechnological protocols exhibiting unique antimicrobial, antioxidant, and anti-inflammatory properties
Source: Front Bioeng Biotechnol. 2026 Apr 15;14:1743887. doi: 10.3389/fbioe.2026.1743887 (PMC13124941; doi:10.3389/fbioe.2026.1743887)
Supplement: Supplementary file 1 [file Table1.docx]

Supplementary Material

Phytosynthesis of Ag@AgCl nanoparticles using two types of bionanotechnological protocols exhibiting unique antimicrobial, antioxidant, and anti-inflammatory properties

Yoshinee Doongoor ^1^, Joyce G Soulange ^1*^, Marek Kolenčík ^2*^

^1^Faculty of Agriculture, University of Mauritius, Reduit 80837, Mauritius

^2^Institute of Agrochemistry and Soil Science, Faculty of Agrobiology and Food Resources, Slovak University of Agriculture in Nitra, Tr. A. Hlinku 2, 949 76 Nitra, Slovak Republic

Table 7 (Supplementary data). Comparative evaluation of anti-inflammatory activity based on BSA inhibition (%) for HE and ME extracts, phytosynthesized Ag@AgCl-NPs, and the reference standard diclofenac sodium, expressed as IC50 values.

| Sample | Equation of line | EC50 (µg/ml) |
| --- | --- | --- |
| Ascorbic acid | y = 0.0676x + 40.725 | 137.20 |
| Heat extract (HE) | y = 0.0734x + 40.574 | 128.42 |
| HE Ag@AgCl-NPs | y = 0.0451x + 21.668 | 628.20 |
| Microwaved extract (ME) | y = 0.073x + 38.3 | 160.27 |
| ME Ag@AgCl-NPs | y = 0.046x + 22.064 | 607.30 |

Table 8 (Supplementary data). Comparison of EC_50_ values for antioxidant activity (DPPH assay) among HE and ME extracts, phytosynthesized Ag@AgCl-NPs, and the standard antioxidant and ascorbic acid as standard reference control.

| Sample | Equation of line | EC50 (µg/ml) |
| --- | --- | --- |
| Diclofenac sodium | y = 0.0689x + 20.816 | 423.57 |
| Heat extract (HE) | y = 0.0691x + 18.276 | 459.10 |
| HE Ag@AgCl-NPs | y = 0.0787x + 24.683 | 321.70 |
| Microwaved extract (ME) | y = 0.0682x + 9.9984 | 586.53 |
| ME Ag@AgCl-NPs | y = 0.0788x + 20.513 | 374.20 |
